# Growing pig incidence rate, control and prevention of porcine epidemic diarrhea virus in a large pig production system in the United States

**DOI:** 10.1186/s40813-022-00268-9

**Published:** 2022-06-07

**Authors:** Mariana Kikuti, Donna Drebes, Rebecca Robbins, Luc Dufresne, Juan M. Sanhueza, Cesar A. Corzo

**Affiliations:** 1 Veterinary Population Medicine Department, University of Minnesota, Saint Paul, MN USA; 2 Veterinary Services, Seaboard Foods, Guymon, OK USA; 3 Departamento de Ciencias Veterinarias Y Salud Pública, Facultad de Recursos Naturales, Universidad Católica de Temuco, Temuco, Araucanía Chile; 4 Previously employed by Seaboard Foods and is currently a swine industry consultant, Amarillo, TX USA

**Keywords:** Porcine epidemic diarrhea virus, Epidemiology, Incidence, Prevention and control

## Abstract

**Background:**

In 2013, PEDV was introduced in the United States (U.S.) and rapidly spread across the country. Here we describe the occurrence of PEDV in the growing pig herd of one large U.S. production system through an active surveillance set in place between October 2019 and November 2020 designed to assess disease status upon placement into the growing pig site, before shipping to the slaughter plant and when diarrhea events were present at the site. We also assessed the impact of preventive procedures implemented in PEDV incidence that comprised site-specific equipment segregation and biosecurity changes regarding personnel movement between sites.

**Results:**

36.50% (100/274) of the sites had at least one PEDV introduction event before preventive procedures were implemented, yielding an incidence rate of 2.41 per 100 farm-weeks. Most (63/100) of them occurred in sites where animals were placed negative and PEDV was detected in clinical samples in a median of 8 weeks post placement. After preventive procedures were implemented, the overall PEDV incidence rate dropped to 0.37 per 100 farm-weeks (84.65% reduction, *p* < 0.001).

**Conclusion:**

These results highlight the importance of systematic surveillance to identify the burden of diseases, areas of improvement in prevention and control, and to allow the measurement of the impact of policy/protocol changes.

## Background

Porcine epidemic diarrhea virus (PEDV) is an RNA, single-stranded, enveloped virus belonging to Alphacoronavirus genus within the *Coronaviridae* family. This virus is responsible for gastrointestinal disease in pigs. It causes an atrophic enteritis of the intestine, which leads to malabsorption and consequently watery diarrhea together with occasional vomiting. In newborn and suckling piglets, the virus causes high morbi-mortality that can reach up to 100%. In older pigs, the disease is mostly manifested by acute transient diarrhea with high morbidity and low mortality. However, poor growth, feed conversion and slight increase in mortality has been reported in growing pigs [1].

This virus has been present in the European and Asian continents for several decades. In 2013, PEDV, characterized both as genogroup 1b (S INDEL) and genogroup 2b (non-S INDEL), was introduced and detected in the United States (U.S.) swine population [2–6]. Rapidly, the virus spread and it was detected in pig farms across the country [7, 8]. A year after its introduction, the virus had been detected in at least 12 states including the main pig producing areas [9]. Unfortunately, PEDV was able to rapidly infect approximately 50% of the breeding herds in the U.S. between 2013 and 2014. Thereafter, the breeding herd PEDV cumulative incidence after 2014 has remained below 10% as prevention, control and elimination strategies were introduced [10]. In fact, a large study that assessed the time needed to control and eliminate this virus from 429 breeding farms concluded that herds required a median time of 28 weeks to successfully achieve this goal [11]. The rapid dissemination and impact of the virus in the first two years after its initial introduction led to shortages in the pig production supply chain, which caused market price fluctuations [12].

The main transmission route for this pathogen is fecal–oral, as large quantities of viral particles are shed into the environment through feces from infected pigs [6]. Alternatively, pigs can become exposed to the virus through PEDV contaminated fomites as viral particles can remain infectious for at least 20 days on objects made out of styrofoam, metal or plastic [13]. An example of fomite contamination aiding transmission is market pig transport vehicles. PEDV positive pigs can contaminate trailers and when arriving to the harvest plant, plant employees’ boots can help disseminate the virus through alleys. These alleys in turn can then serve as a source of virus for incoming clean trucks that get contaminated during the pig unloading process [14]. In fact, time of marketing was one of the main causes for enteric coronaviruses introduction in finishing sites of a large system between November 2018 and March 2019 [15]. Movement of positive pigs represents a risk not only because it contaminates transport vehicles, but also because it has been implicated in the regional dissemination of the virus when pigs are moved between production stages [16]. This can also contribute to local area spread as the virus can become airborne [17]. By better understanding its behavior, infection dynamics in the growing pig herd and how it is transmitted between growing pig sites, control and prevention measures can be developed to decrease the number of actively shedding pigs.

Here, we summarize the occurrence of PEDV in the growing pig herd of one large U.S. production system through an active surveillance together with the impact that preventive procedures implemented had on disease occurrence. The specific objectives of this study were: (a) to quantify the number of PEDV introductions per geographic region to further understand regional burden of the disease, (b) to determine the time frame of PEDV introductions in relation to the placement of animals, and (c) to decrease occurrence through the implementation of preventive intervention strategies.

## Results

### Pre-intervention testing results

**Fig. 1:**
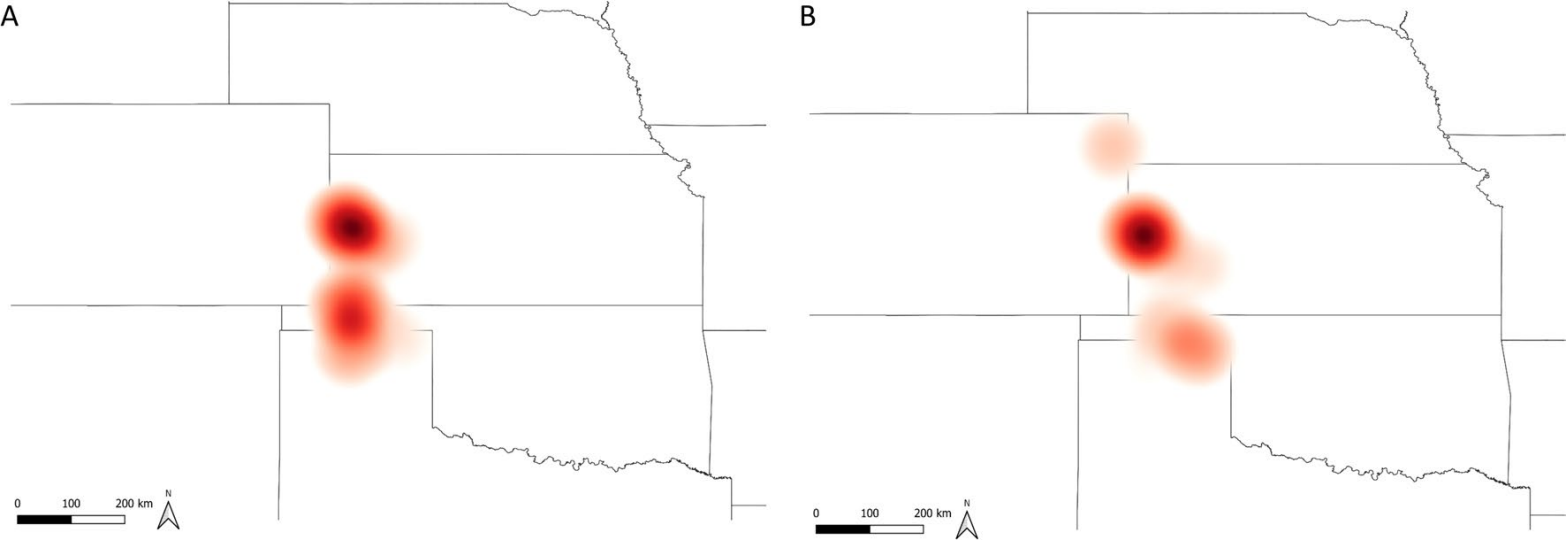
Kernel density map of PEDV introductions during the pre- **A** and the post-intervention **B** periods

About a third of the sites (36.50%; 100/274) had at least one PEDV introduction event during the first 21 weeks of study. Out of those, almost all of them (99/100) had only one introduction event, whereas one site had two introduction events (1/100). Incidence rate overall and by geographic region for the first 21 weeks of the study (pre-intervention period) are described in Table 1. Overall, the farm-level PEDV incidence rate during the first 21 weeks of surveillance was 2.41 per 100 farm-weeks, meaning this system experienced an average of almost three PEDV introductions for every 100 farms monitored in a week. During this period, most introductions occurred in western Kansas and Oklahoma (Fig. 1A).

**Table 1:** Number of sites and incidence rate by PEDV introduction events, geographical region and intervention timeframe

Geographic region	Pre-intervention					Post-intervention				
	Number of PEDV introductions			Farm-weeks at risk	Incidence rate per 100 farm-weeks (95%CI)	Number of PEDV introductions			Farm-weeks at risk	Incidence rate per 100 farm-weeks (95%CI)
	0	1	2			0	1	2		
A	26	22	0	698	3.15 (2.09–4.73)	48	0	0	1297	0.00 (0.00–0.30)
B	8	1	0	118	0.85 (0.15–4.64)	6	3	0	285	1.05 (0.36–3.05)
C	42	8	0	916	0.87 (0.44–1.71)	47	3	0	1695	0.18 (0.06–0.52)
D	8	0	0	160	0.00 (0.00–2.34)	8	0	0	296	0.00 (0.00–1.28)
E	18	14	0	543	2.58 (1.54–4.28)	31	1	0	916	0.11 (0.02–0.62)
F	13	5	0	248	2.02 (0.86–4.63)	13	5	0	509	0.98 (0.42–2.28)
G	13	9	0	318	2.83 (1.50–5.29)	21	1	0	648	0.15 (0.03–0.87)
H	3	5	0	118	4.24 (1.82–9.54)	7	1	0	209	0.48 (0.08–2.66)
I	14	21	1	548	4.20 (2.81–6.22)	32	4	0	1175	0.34 (0.13–0.87)
J	15	9	0	297	3.03 (1.60–5.66)	17	6	1	792	1.01 (0.51–1.98)
K	14	5	0	228	2.19 (0.94–5.03)	15	3	1	534	0.94 (0.40–2.17)
Total	174	99	1	4192	2.41 (1.99–2.92)	245	27	2	8356	0.37 (0.26–0.53)

PEDV: Porcine epidemic diarrhea virus; 95%CI: 95% confidence interval

Most (63/100) of those introductions occurred in sites in which a negative post placement sample had been obtained followed by positive results in clinical samples. Unfortunately, on two cases, results from the samples involved in the status change were missing. It is likely that this site was classified as positive due to a sister site (< 1000 feet, or approximately 305 meters) testing positive. For two additional cases, a post placement test result was missing, so no samples involved in the status change were attributed. Those four sites were excluded from the analysis regarding timing of introduction. Frequency of each of the combinations of latest negative to positive sample types and the time in weeks between both are described in Table 2. Although a large variability in time was observed, negative post placement samples to positive clinical samples had a median of eight weeks interval, whereas negative pre shipment samples to positive clinical samples had a median of two weeks interval.

**Table 2:** Timeframe of PEDV introductions in the pre-intervention period

Previous negative sample	Positive sample	n	%	Weeks between samplings
				Median (IQR)
Clinical	Pre shipment	1	1.03	1
Post placement	Clinical	65	67.01	8 (6–11)
Post placement	Pre shipment	7	7.22	16 (10–19)
Pre shipment	Clinical	6	6.19	2 (2–2)
Pre shipment	Post placement	18	18.56	4.5 (4–6)

*PEDV* Porcine epidemic diarrhea virus, *IQR* interquartile range

### Post-intervention testing results

The overall PEDV incidence rate in the 37 weeks post-intervention was 0.37 per 100 farm-weeks (less than one PEDV introduction for every 100 farms monitored in a week) (Table 1). This represents an 84.65% reduction in the PEDV incidence rate compared to the pre-intervention period (chi-square *p* < 0.001). PEDV incidence rate was also smaller in the post-intervention period compared to the pre-intervention period when accounting for farm and location (HR = 0.01, 95%CI 0.002–0.02, *p* < 0.001). The region with higher PEDV occurrence during this period was western Kansas (Fig. 1B).

## Discussion

Infectious diseases continue to represent an important burden to the swine industry. The current study has shown how a large production system controlled and prevented transmission of PEDV through the integration of a surveillance program, data analysis and biosecurity program adjustments. To the authors knowledge, this is the first to report addressing PEDV control and prevention in growing pigs. This directly complements the efforts made at the breeding herd level, which ultimately leads to contributing to regional biosecurity.

The results from the surveillance program implemented in this case demonstrated that most of the growing pig sites remained PEDV negative once the post-placement sample was collected. Such finding is important because it emphasizes two important epidemiological pieces that are required to control and prevent PEDV in growing pigs. The first one being the confirmation of the PEDV negative status of the pigs arriving to the site. Even though these pigs were born to PEDV free sows, potential PEDV exposure could have occurred at any given time during transport hindering these efforts. Secondly, the cleanliness of the building where pigs arrived to was confirmed as pigs acted as sentinels. This finding contributed to break a paradigm in that at some point it was thought that facilities were not being cleaned appropriately. If infectious PEDV particles were to be present, diarrhea would have started shortly after arrival. Therefore, the surveillance program contributed to fine tuning these processes that lead to ensuring a high biosecurity standard. Viral introductions into these PEDV negative growing pig sites still occurred throughout the study period. However, as characterized, these occurred on average eight weeks after placement. It is unlikely that these introductions represented undetected infections during and after placement considering a PEDV incubation period of two to four days [18]. For these reasons, a review of the biosecurity protocols in the growing pig herds was initiated to reduce viral introductions in this population.

Daily and weekly communication of growing pig site PEDV status within the company through test results and farm status mapping allowed field personnel to plan their farm visits accordingly and thus reduce the risk of between farm transmissions as positive sites would be visited last. However, it was not until changes in specific procedures were implemented such as segregation of equipment and personnel entry protocols that there was a reduction in the incidence. These procedural changes led to an important investment in equipment that was certainly proved efficient for PEDV but may have also contributed to restricting transmission of other pathogens.

An important limitation of this study is the fact that no temporal control was designated to assess if the reduction in incidence was impacted by factors other than the intervention. However, PEDV cumulative incidence in breeding herds in the production system and the U.S. has remained substantially the same throughout the 2018–2019 and 2019–2020 seasons [10], suggesting the expected PEDV incidence during the time frame of this study should be similar pre- and post-intervention. Additionally, co-occurrence of other enteric coronaviruses was not assessed. However, the RT-PCR used to detect PEDV is indeed a multiplex testing PEDV, porcine deltacoronavirus (PDCoV), and transmissible gastroenteritis virus (TEGV) simultaneously. No TGEV has been detected in sites pertaining to this system and, although PDCoV have been detected sporadically, PDCoV results did not influence the recording of PEDV site statuses.

## Conclusion

An active surveillance detected a PEDV incidence of 2.41 per 100 farm-weeks in this growing pig population with most of the PEDV introductions occurring around eight weeks after placement. This insight resulted in a segregation of equipment intervention strategy that seemed to have reduced the PEDV incidence in over 80%. This highlights the importance of systematic surveillance to identify the burden of diseases, areas of improvement in prevention and control, and to allow the measurement of the impact of policy/protocol changes.

## Methods

### Studied population

This study was conducted using data from a large U.S. vertically integrated multi-site pig production system with live operations in five states throughout the country. The pig production system through their health veterinary team oversees the biosecurity, health and production aspects of commercial pig production, live-animal transport, cleaning-disinfection and feed manufacturing. Their breeding farms wean piglets approximately at 19 days of age (DOA) into off-site all-in all-out by barn (few sites that are completely empty before placing new pigs due to space constraints) nurseries where pigs remain approximately for six to seven weeks. Once pigs are approximately 63–67 DOA they are transported to all-in all-out by site premises (e.g. finishing farms) in 11 regions located in four states until they reach market weight. Each growing pig site is composed of multiple pig barns ranging from three to 20 barns and managed by regional pig care professionals who oversee the daily operations of the farm and ensure that pigs are cared for and remain healthy. Growing pig site personnel biosecurity includes the use of a shower-in and use of farm specific clothing and boots. No UV-chambers or disinfection and drying rooms are used in any of these sites. Rendering is used from a mortality management standpoint in which a truck and a trailer would go farm by farm collecting the mortality and bring it to a central collection point.

At the pig transport level, the company has designed a process to mitigate infectious disease risk in the growing herd. Recently weaned pigs are transported from the sow farm to the nurseries in trailers that have undergone cleaning, washing, and thermo-assisted drying and decontamination (TADD) at a minimum of 71 °C for 15 min. The first load of feeder pigs transferring on any given day from the nursery to the finisher will have a cleaned, washed and TADD trailer. Turning around a trailer is only allowed when both the nursery and the finisher are PEDV negative. If transfer into a PEDV positive site, that trailer will only be used to move animals between PEDV positive sites until it is cleaned, washed, and decontaminated using a TADD. Shipments to the packing plant or to third party aggregators are done with a dedicated trailer fleet. At the finishing site, once pigs are ready to be marketed, a trailer that had been cleaned, disinfected, and that had gone through the TADD system would load the first group of pigs. Remaining loads of pigs will be loaded on a truck that has not gone through cleaning and disinfection as these sites are to be emptied within a week.

The production company has six feed mills constantly providing feed to all farms within the production system. Trucks and feed trailers are washed once a week and a one-night downtime is specifically required for those trucks needing to go from a positive to a negative health status site in the case of boar studs, sow farms and gilt development units. From November 19th, 2019, onwards, the driver delivering feed would only get off the tractor-cab once a pair of plastic botties were on the drivers’ shoes. During the study period, the production system was not including any kind of infectious disease feed mitigants.

The company was part of the large PEDV outbreak in the country as a total of 36 and 28 out of 63 breeding herds became infected in 2013 and 2014, respectively. The veterinary team worked diligently to rapidly control and eliminate PEDV from their breeding herds. Briefly, once the veterinary team collected samples, submitted them to the Veterinary Diagnostic Laboratory and received confirmation that the herd was PEDV positive, the team followed a similar protocol used to eliminate other viral diseases (e.g. Porcine Reproductive and Respiratory Syndrome). The protocol implemented is known as load-close-expose which basically refers to introducing into the herd as many replacement gilts as possible, stop further introductions and homogenize the PEDV status of all the population of breeding animals. The overall goal of this protocol is to rapidly generate herd immunity so that neonatal pigs have access to PEDV immunity via colostrum intake. Furthermore, through this process the duration of population shedding is shortened that when coupled with strict cleaning, disinfection and internal biosecurity protocols, elimination of the virus is attained leading to weaning PEDV PCR negative piglets [18].

### Background, active surveillance, and herd classification

Given that the company continued to experience PEDV cases with a median of 7.5 breeding sites (interquartile range (IQR): 4 – 11) affected yearly in 2015–2018, the company started a PEDV surveillance program in the nursery in early 2018 to determine status of company nursery sites. During 2019, the company extended the surveillance to the finishers after most nursery sites were known PEDV negative. The surveillance program implemented in both nursery and finishing sites allowed them to identify patterns of disease occurrence and areas for potential improvement, with the ultimate goal of reducing the PEDV burden across the whole pig production system.

The active surveillance was designed to understand when PEDV introductions were occurring regarding placement of the animals in the 274 growing-finishing sites involved in this study between October 13, 2019 (calendar week 42 of 2019) and November 22, 2020 (calendar week 47 of 2020) (Fig. 1A). The surveillance sampling protocol was designed to assess disease status (1) upon placement into the growing pig site, (2) before shipping to the slaughter plant and (3) when diarrhea events occurred at the site. Briefly, upon pig placement, one composite Swiffer sample was collected one week after the site had been fully filled. This composite sample was the result of rubbing the Swiffer on an area of one meter by one meter of the slatted flooring within a meter of the feeder of two designated pens from all barns on-site. The pre-shipment sampling was one oral fluid [19] sample per barn one week before the site started to ship pigs to market. If diarrhea was observed in these growing pigs, one composite Swiffer was collected. All samples were RT-PCR tested at the Iowa State University Diagnostic Laboratory. Test results yielding a cycle threshold (Ct) below 30 were considered positive. However, if the Ct value yielded was 30 or above and there was absence of clinical signs, the test results would be classified as negative since detectable viral particles could be the result of remanent non-infectious genetic material from the previous group. The interpretation was based on previous experience with RT-PCR testing and together with discussions with laboratory diagnosticians.

Once diagnostic test results were available, farm health statuses were updated daily for each farm according to the combination of the latest test results. In addition, a system-wide message was sent immediately if a positive test occurred in a boar, sow or gilt development unit and no later than the next morning if a growing pig site had yielded a positive test result. If two sites that were close together shared employees and one of them tested positive, the negative site would automatically be classified as positive. For the purposes of this study, a farm’s status was considered negative when the most recent test (either pre-shipment or post-placement) yielded a negative PCR result (Ct > 30) and no clinical signs (i.e. diarrhea) were observed. A virus introduction was defined when a site that had tested negative in the most recent test (pre-shipment or post-placement) then tested positive in pre-shipment, post-placement, or when diarrhea events occurred. Weekly status of each farm was mapped using Leaflet R package [20, 21] and timely shared with the field personnel through an html file to allow the planning of their field activities accordingly. Briefly, farm personnel in-charge of multiple sites and regional growing pig site supervisors organized their farms visits according to status (e.g. PEDV negative sites were visited at the beginning of the day leaving positive farms towards the end of the day to avoid potential cross-contamination).

### PEDV incidence rate and time frame between placement and virus introduction

We considered a PEDV introduction whenever there was a change in weekly status from negative to positive. Farm-level incidence rate was calculated by dividing the total number of PEDV introductions by the total amount of weeks monitored in which farms were followed but did not have a positive status. Incidence rate overall and by production region was described for the first 21 weeks of study (pre-intervention period) and the last 37 weeks (post-intervention period). Time in weeks between the last negative sample and the positive sample responsible for the status change in the event of a PEDV introduction were calculated by sample types involved (pre shipment sampling – PST, post-placement sampling – PL, sampling when clinical signs were observed – CS).

### Intervention strategy

With the findings from the first 21 weeks of surveillance enough data was generated to propose an intervention strategy to reduce PEDV incidence in the monitored sites. The intervention consisted of a system-wide segregation of farm equipment, facility cleaning and disinfection processes improvement and the inclusion of a hydrogen peroxide-based disinfectant (Decon 7, Coppell, TX, USA) after the 21st week. Briefly, the company decided to invest in site-specific equipment with the goal of segregating sites and mitigate indirect transmission through contaminated fomites. Therefore, pig sorting boards, shaker cans, necropsy tools, small step ladders, buckets, hoses, brooms, snares, notebooks, wrenches, screw drivers and dead haul trailers were purchased for each site. Mobile loading chutes used during market hog loading are now washed and baked to avoid between-farm cross contamination. Furthermore, internal biosecurity protocols regarding personnel movement between sites (e.g., traveling clothes, bench at the entry separating dirty from clean areas at each site, changing into farm clothes, dirty coveralls not allowed in cab of truck) were reviewed and emphasized with staff. Employees were asked to wear gloves when handling pigs together with sanitizing hands (i.e. at minimum application of alcohol-based sanitizer until dry or, when available, washing hands using soap and water for 20 s) after exiting the barn and before traveling to another farm. The active surveillance continued uninterrupted without any changes after the intervention, which allowed a comparison of its effect in PEDV incidence. Overall PEDV incidence rates pre- and post-intervention were compared by chi-square. Additionally, a cox proportional hazards model was used to estimate the hazard ratio (HR) between the pre- and post-intervention number of outbreaks accounting for geographic region as a fixed effect, farm as a random effect, and for time at risk as the offset.

### Geographic distribution

The point location of all but 16 sites was obtained. A kernel density map (i.e. heatmap) showing PEDV introductions in the period before and after the intervention strategy was constructed using a radius of one map unit in QGIS v3.14.16-Pi [22].

## Abbreviations

PEDV: Porcine epidemic diarrhea virus
U.S.: United States
RNA: Ribonucleic acid
HR: Hazard ratio
95%CI: 95% confidence interval
DOA: Days of age
UV: Ultraviolet
TADD: Thermo-assisted drying and decontamination
PCR: Polymerase chain reaction
IQR: Interquartile range
RT-PCR: Reverse transcription polymerase chain reaction
Ct: Cycle threshold
PST: Pre shipment sampling
PL: Post-placement sampling
CS: Clinical signs
PDCoV: Porcine deltacoronavirus
TEGV: Transmissible gastroenteritis virus

## Declarations

### Availability of data and materials

The dataset used is privately owned by the company. Data are, however, available from the authors upon reasonable request and with permission of Seaboard Foods but restrictions might apply.
